# Characterization of rapid neutrophil extracellular trap formation and its cooperation with phagocytosis in human neutrophils

**DOI:** 10.15190/d.2014.11

**Published:** 2014-06-30

**Authors:** Mona Saffarzadeh, Hector A. Cabrera-Fuentes, Florian Veit, Dongsheng Jiang, Karin Scharffetter-Kochanek, Christian Gille Gille, Suzan H. M. Rooijakkers, Dominik Hartl, Klaus T. Preissner

**Affiliations:** ^1^Department of Biochemistry, School of Medicine, Justus-Liebig-University, Giessen, Germany; ^2^Center for Thrombosis and Hemostasis (CTH), University Medical Center Mainz, Mainz, Germany; ^3^Excellence Cluster Cardio-Pulmonary System (ECCPS), German Lung Center (DZL), Justus-Liebig-University Giessen, Giessen, Germany; ^4^Department of Dermatology and Allergic Diseases, University of Ulm, Ulm, Germany; ^5^Department of Pediatrics IV/Neonatology, Eberhard Karls University, Tuebingen, Germany; ^6^Medical Microbiology, University Medical Center Utrecht, Utrecht, The Netherlands; ^7^Department of Pediatrics I, Eberhard Karls University, Tuebingen, Germany

**Keywords:** Rapid NETosis, Neutrophil extracellular traps (NETs), Phagocytosis

## Abstract

Neutrophils, as the first cellular line of innate host defense, employ phagocytosis and formation of neutrophil extracellular traps (NETs) to combat infections. Classical NET formation induced by phorbol myristate acetate requires several hours to complete. However, recent studies demonstrated rapid NET formation in neutrophils upon stimulation by platelets, Staphylococcus aureus or fungal products. Here we describe that antibody- or complement-induced phagocytosis triggers rapid NET formation. In contrast to classical NETosis, chemical inhibition of NADPH oxidase as well as using NADPH oxidase-deficient patient neutrophils did not affect rapid NET formation. Although phagocytosis and rapid NET formation may not be the prerequisite of each other, cooperation of phagocytosis and rapid NET formation may be essential to improve the efficiency of defense mechanisms in combating disseminating bacteria. Dissecting the differential mechanisms of NET formation is crucial to develop novel therapeutic strategies for infectious and auto-immune diseases where NETs play an essential role.

## INTRODUCTION

Neutrophils form the first cellular line of host defense against microbes. To combat infection, neutrophils apply several antimicrobial mechanisms, including the release of anti-microbial peptides and lytic enzymes from intracellular granules^1-3^. Since neutrophils are professional phagocytes, they engulf microbes at the site of infection into phagosomes: Following fusion with neutrophil granules, which discharge their antimicrobial contents into the lumen of the phagosome, phagolysosomes are generated, where the pathogens are killed after exposure to enzymes, antimicrobial peptides and reactive oxygen species (ROS). The latter are produced by the nicotinamide adenine dinucleotide phosphate oxidase (NADPH oxidase) complex (assembled at the phagosomal membrane). Finally, phagocytosed bacteria are killed by this combination of antimicrobials and ROS^4-8^.

Foreign particles need to be opsonized with complement or antibodies in order to be effectively phagocytosed by neutrophils. The key molecules mediating phagocytosis of opsonized particles are complement and Fc receptors^8,9^. Particles coated with IgG antibody on their surface have the Fc region exposed that binds to the corresponding receptor on the cell surface and enhances phagocytosis. Besides Fc receptors, phagocytic cells express two receptors designated CR1 (CD35) and CR3 (Mac-1, α_M_β_2_ or CD11b/CD18), which binds the third component of the complement system C3b. Binding of C3b-coated particles to these receptors results in enhanced phagocytosis and stimulation of the respiratory burst^6,8,9^.

A more recently appreciated antimicrobial mechanism of neutrophils is mediated via formation of neutrophil extracellular traps (NETs)^10^, where neutrophils eject their own chromatin content mixed with granular components^11^. This DNA/histone scaffold allows trapping of microorganisms and facilitates their killing by the highly concentrated antimicrobial components. In addition to their antimicrobial activity, NETs also cause host cell cytotoxicity, promoted mainly by the histone components, and may thus be involved in tissue destruction^12,13^. Gram-positive or -negative bacteria, fungi, protozoa, activated platelets as well as the protein kinase C activator phorbol myristate acetate (PMA) were described to activate neutrophils to form NETs^14-17^.

In response to PMA, the kinetic process of NETosis is induced whereby neutrophils undergo several morphological changes: loss of nuclear and granular integrity, chromatin decondensation, merging of chromatin and granular/cytoplasmic proteins as well as disintegration of the plasma membrane. Release of NETs and chromatin extrusion in response to PMA start after 1 h, which is fully dependent on NADPH oxidase activity, with a maximal response after approximately 3 h^16^. However, recent studies demonstrated rapid NETosis completed in less than 1 h: NETs were found to be generated within minutes after activation by stimulated platelets under flow^15-18^, or in response to fungal product in the presence of extracellular matrix components^19^. Moreover, rapid NET formation in response to *Staphylococcus aureus* appears to occur within minutes in an NADPH oxidase-independent manner^20^. Also a recent study has revealed that during skin infection in mice, bacteria can induce phagocytosis, while simultaneously triggering rapid NET formation within minutes^21^. Yet, the interplay between phagocytosis inducers and NETosis and the underlying mechanisms in human neutrophils are not defined. In this study, we report on a cooperative effect between phagocytosis and NETosis, whereby typical phagocytosis inducers trigger rapid NET formation.

## MATERIALS AND METHODS

### Ethics statement

All healthy individuals or patients gave a written informed consent, approved by the Ethics Committees of the Medical Faculties, of the Justus-Liebig-University, Giessen and the Eberhard-Karls-University, Tuebingen (file numbers 05/00 and 178/2011BO1).

### Isolation of human neutrophils

Human peripheral blood neutrophils were isolated from healthy donors or chronic granulomatous disease (CGD) patients as previously described^22^. Briefly, a double gradient was formed by layering an equal volume of histopaque-1077 over histopaque-1119 (Sigma-Aldrich, Germany). Venous blood was carefully layered onto the upper histopaque-1077, followed by centrifugation at 700 g for 30 min and granulocytes were concentrated at the 1077/1119 interphase. The remaining erythrocytes were lysed by hypotonic shock using distilled water, and neutrophils were resuspended in phenol red-free RPMI 1640.

### Phagocytosis assay

Phagocytosis assay was performed using latex beads-rabbit IgG-FITC complex (Cayman Chemical, Germany) or fluorescent-labeled C3b-coated beads. The latex beads had a 0.1 µm mean particle size. The beads were supplied from the manufacturer as a weight to volume percentage. The labeling and aliquoting were done on a weight or volume basis, not on the bead count and they were unmodified polystyrene. The Streptavidin fluoresbrite microspheres (Polysciences) which were used for C3b coating had 2.0 μm particle size. Based on the manufacturers, these particles were packaged as 1.25% solids in aqueous suspension. To prepare the fluorescent-labeled C3b-beads, 10 µl of streptavidin fluoresbrite microspheres in 1 ml PBS/0.1% BSA were incubated with site-specifically (thioester) biotinylated C3b^23^ at the final concentration of 1 µg/ml for 60 min at 4^o^C. Microspheres which were incubated only in PBS/0.1% BSA without C3b were used as control beads. After washing, the beads were resuspended in 300 µl RPMI medium with 0.05% HSA. For each condition, 10^5^ neutrophils in 300 µl RPMI were incubated with 10 µl of latex IgG-beads (dilution 1:40), with 20 µl of C3b or with control beads for 10, 30, 60 and 150 min at 37°C with 5% CO_2_. To inhibit Fc-dependent phagocytosis, neutrophils were pre-treated for 30 min with 20 µg/ml of the recombinant staphylococcal protein FLIPr-like protein. Percentage of fluorescent labeled beads per total number of cells was considered as phagocytosis.

### Immunofluorescence microscopy

For each condition, 10^5^ neutrophils were seeded on coverslips and treated with 25 nM PMA (Sigma-Aldrich), IgG-beads, C3b- or control beads, from 10 min to 150 min at 37°C with 5% CO_2_. For inhibitory experiments, neutrophils were pre-treated for 30 min with 20 µg/ml FLIPr-like protein, 20 µM Cytochalasin D (CytD), 10 µM Diphenyl-eneiodonium chloride (DPI) or DMSO as vehicle. To visualize NETs the samples were fixed with 1% paraformaldehyde (PFA), blocked with 3% bovine serum albumin (BSA) and incubated with primary mouse anti-DNA Histone H1 (Millipore, Germany) followed by detection with secondary antibody coupled to Alexa Fluor 555 donkey anti-mouse IgG (Invitrogen). This DNA/histone antibody has a very high affinity for decondensated chromatin in NETs in comparison to DAPI or Hoechst as it was shown before^12^. Hoechst 33342 (Invitrogen) was used for nuclear DNA detection. Slides were mounted with ProLong Gold antifade reagent (Invitrogen). Cell images were taken with fluorescence microscope (Leica Microsystems, Wetzlar, Germany), and NETs were quantified based on the area of DNA/histone antibody per number of cells detected by Hoechst. MetaMorph imaging software version series 7.0 (Leica Microsystems, Wetzlar, Germany) was used for NET quantification.

### Electron paramagnetic resonance measurements

Human neutrophils were normalized to 10^5^ cells per sample, incubated with IgG-beads or PMA, and ROS production of treated samples was compared to untreated controls. ROS release from neutrophils was measured as described with modifications^24^. Briefly, electron paramagnetic resonance (EPR) measurements were performed at -170°C using an MS 100 ESR spectrometer (Magnettech, Berlin, Germany). We used the spin probes CMH (1-hydroxy-3-methoxycarbonyl-2,2,5,5-tetramethyl-pyrrolidine) and CPH (1-Hydroxy-3-carboxy- 2,2,5,5-tetramethylpyrrolidine) (500 µM) for the detection of intra and extracellular (CMH) or only extracellular (CPH) ROS production by neutrophils. CMH or CPH were added to the samples for 20 min, shock frozen and stored in liquid nitrogen until measurement. The spectrometer settings were as follows: g-factor = 2.0063, center field = 3349.95 G, microwave power = 200 mW, sweep time = 20 sec, sweep number = 5.

### Statistical analyses

Data were analyzed by GraphPad Prism 5.03 software using one-way analysis of variance (ANOVA) with Tukey post-tests for multiple comparisons or by unpaired, two-tailed *t* test for single measurements. Reported values are expressed as mean ± s.e.m. Each experiment was performed at least three times on independent occasions unless otherwise stated. Differences were considered statistically significant at p<0.05. In the figures, significant differences were illustrated with asterisks (*p<0.05; **p<0.01; ***p<0.001).

## RESULTS AND DISCUSSION

### IgG-beadstrigger and accelerate NET formation

**Figure 1:**
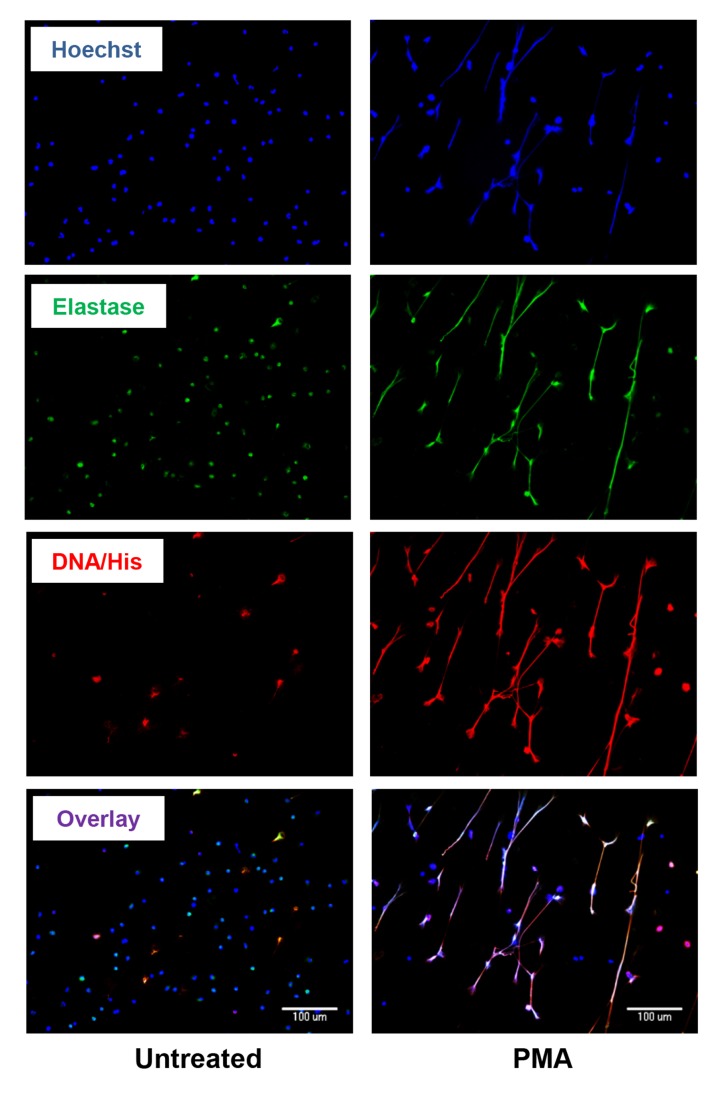
NET characterization by immunocytochemistry Human neutrophils were kept untreated or treated with PMA for 150 min. Immunohistochemistry was performed to visualize NETs using DNA Histone antibody (red), elastase (green), and Hoechst 33342 (blue) for nuclear detection. Note that the DNA Histone antibody has a very high affinity for the decondensated chromatin in NETs.

**Figure 2:**
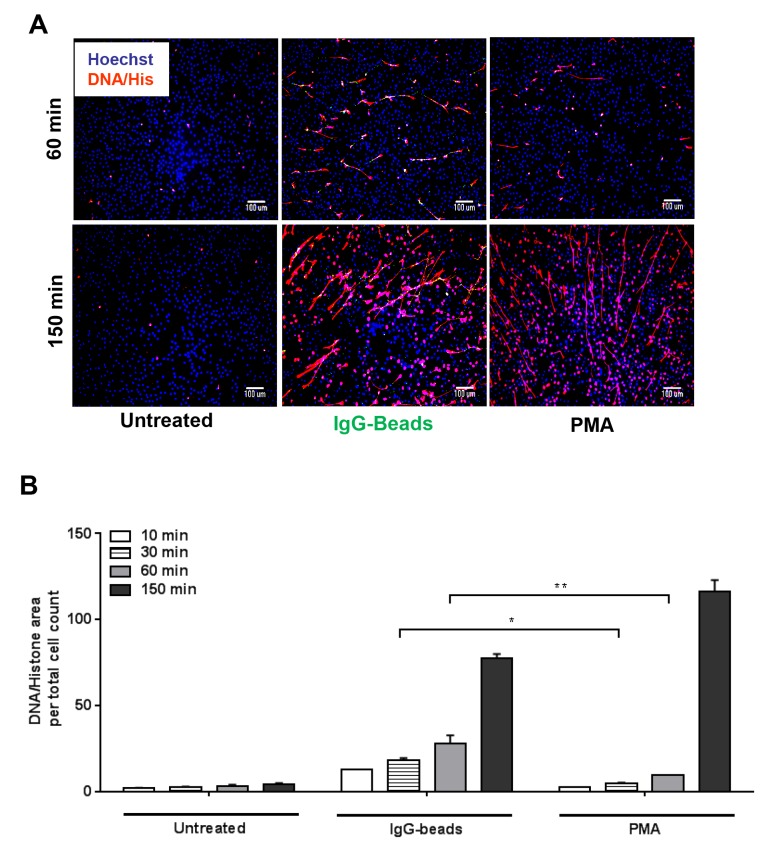
IgG-beads trigger and accelerate NET formation (A) Human neutrophils were kept untreated or incubated with IgG-beads or PMA for 60 min or 150 min, and immunocytochemistry was performed to detect NETosis which is depicted by DNA histone antibody. Nuclear stain: Hoechst 33342. Note that PMA alone did not induce considerable NET formation during 1 h, while IgG-beads accelerate NETosis. (B) Neutrophils were treated with IgG-beads or PMA for 10, 30, 60 and 150 min, and NET formation was quantified by ratio of DNA-histone area per number of cells as analyzed in panel A. Data are representative of at least three independent experiments.

Thorough extrusion of chromatin from neutrophils and NET formation classically requires hours to be completed when the cells are treated with PMA^16^. **Figure 1** shows the fibrous structure of chromatin that appears outside of the cell bodies after stimulation of neutrophils for 150 min with PMA. The decondensed chromatin in NETs is apparent by using a DNA/histone antibody, and another major NET-protein, elastase that is entangled in these fiber-like structures, and further confirms NETs (**Figure 1**). To address the question how phagocytosis and NET formation interact with each other in the same neutrophil population, human neutrophils were incubated with IgG-opsonized beads for 150 min (**Figure 2** [A], lower panel). Since phagocytosis occurs usually within minutes, we wondered whether incubation of beads for a shorter period of time can also trigger NET formation. Analysis of neutrophils by immunocytochemistry revealed that IgG-beads not only induced NET formation, but they were also able to enhance NETosis in a shorter time period (60 min) in comparison to PMA (**Figure 2** [A], upper panel).

The employed DNA/histone antibody^12^ has a very high affinity for decondensated chromatin in NETs in comparison to DAPI or Hoechst stain, similar to the antibody used in the previous study^25^, and can be used for quantification. The appearance of extracellular DNA in unstimulated controls most likely originated from auto-activation of neutrophils during isolation. In all conditions used here, the total number of cells kept equal; therefore, the areas of decondensed chromatin per total number of cells were measured to quantify NETs. Incubation of neutrophils with IgG-beads for even shorter time periods (30 and 10 min) also induced NET formation, while PMA was incapable of inducing NETosis (**Figure 2** [B]). These data indicate that IgG-beads may accelerate NETosis.

### Cytochalasin D reduces both IgG-beads and PMA-induced NETosis

**Figure 3:**
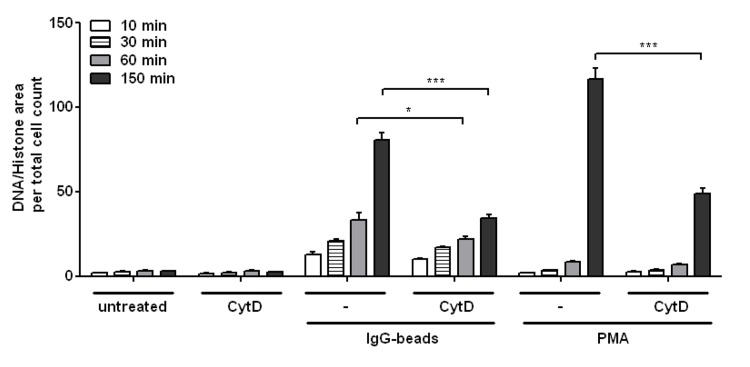
CytD reduces both IgG-beads and PMA-induced NETosis Neutrophils were pre-treated with CytD for 30 min, followed by addition of IgG-beads or PMA for 10, 30, 60 or 150 min. NETosis was quantified by ratio of DNA-histone area per number of cells. Data are representative of three independent experi-ments.

To further investigate the functional relationship between phagocytosis and NETosis, cells were pre-treated with CytD, which is an inhibitor of actin polymerization and internalization of receptor-bound latex beads^26^. Thereafter, the kinetics of PMA as well as IgG-induced NETosis was analyzed. CytD decreased PMA-mediated NET formation (**Figure 3**), which confirms previous observations in similar experimental systems^27,28^. Moreover, CytD also significantly reduced bead-mediated NETosis. Since substantial NET formation after 150 min requires much more intracellular structure reorganization, inhibition of NET formation by CytD maybe more prominent at the later time points. Although CytD is usually used as a blocking reagent in studies dealing with phagocytosis, it is not a specific inhibitor of phagocytosis. Actin polymerization appears to be mandatory for NET formation^29^, which requires massive reorganization of cellular structures to mix the chromatin with granular proteins and expel it from the cell. Therefore, more specific inhibitors of phagocytosis were used in the following experiments.

### IgG-beads and C3b induce both phagocytosis and NETs in a time-dependent manner

**Figure 4:**
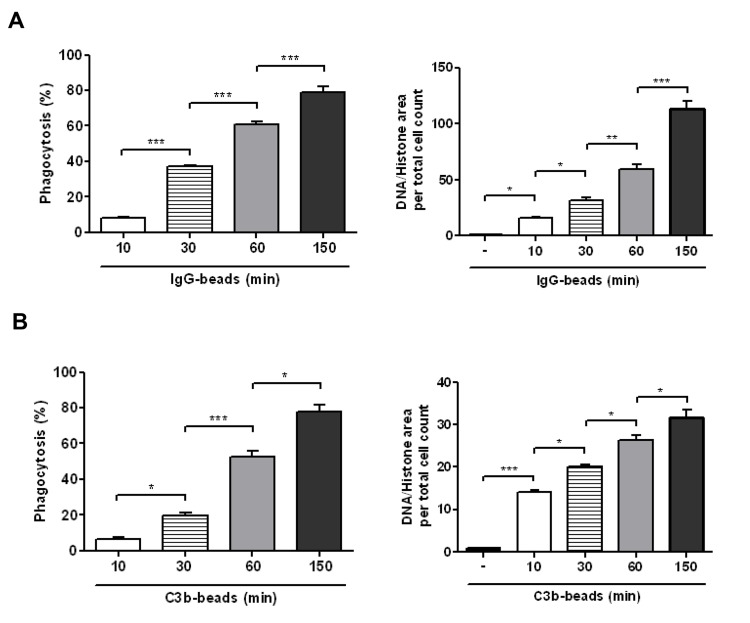
IgG-beads and C3b induce both phagocytosis and NET in a time-dependent manner (A) Neutrophils were incubated with IgG-beads or (B) with C3b-coated beads for 10, 30, 60 and 150 min, followed by quantification of phagocytosis and NETosis. Note that phagocytosis and NET formation increased concomitantly.

In addition to IgG-coated beads, we also used C3b-coated beads as a second independent opsono-phagocytosis inducer. Both IgG- and C3b-coated beads induced phagocytosis in a time-dependent manner (**Figure 4**). Concomitantly, both coated beads increased NETosis in a time-dependent fashion, and as it was observed for IgG-beads, C3b-coated beads were also able to induce NET formation in a short period of time (from 10 min).

**Figure 5:**
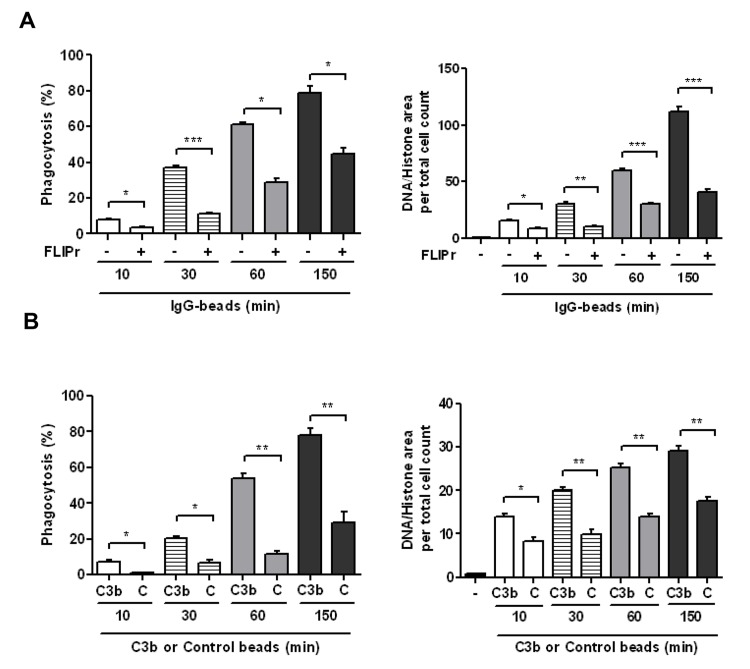
Inhibition of FcR and complement-dependent phagocytosis reduce NET formation (A) Neutrophils were pre-treated with FLIPr-like protein for 30 min; followed by incubation of cells with IgG-beads for 10, 30, 60 and 150 min. Phagocytosis and NETosis were quantified in comparison to the cells without FLIPr pre-treatment. (B) Neutrophils were incubated with either C3b-coated beads or with uncoated beads as controls (depicted in the figure as C) for 10, 30, 60 and 150 min. Thereafter, NET generation and phagocytosis were quantified.

*S. aureus* escapes from FcγR-mediated immunity by secreting a potent FcγR antagonist, FLIPr, or its homolog FLIPr-like^30^. FLIPr-like binds to different FcγR subclasses and blocks IgG-ligand binding, and thus it inhibits FcγR-mediated opsono-phagocytosis. To more specifically inhibit opsono-phagocytosis, we used FLIPr-like protein. Pre-treatment of neutrophils with FLIPr-like protein reduced IgG-beads phagocytosis (**Figure 5** [A]). As expected, when FcγR was blocked with this antagonist, also significant reduction in IgG-beads-induced NET formation was observed (**Figure 5** [A], upper right).

Using control beads, which are not coupled with C3b, both phagocytosis and NET formation were reduced in comparison to when neutrophil are treated with C3b-beads (**Figure 5** [B]). Collectively, these data indicate that NETs and phagocytosis act in a cooperative way.

By incubation of neutrophils with Fc fragments, we could not observe considerable NET formation in comparison to the untreated cells (data not shown). On the other hand, we also assessed phagocytosis and internalization by colocalization of IgG-beads or GFP-E. coli with lysosome-associated membrane protein-1 as well as by phase contrast microscopy, and we could see increase in both phagocytosis as well as early NET formation after incubation of cells with IgG-beads or GFP-E.coli (data not shown). Similarly, incubation of neutrophils with GFP-*E. coli* induced rapid NETosis (**Supplementary Figure [S1]**). Since, in IgG-dependent phagocytosis, there is Fc receptor activation, we cannot rule out the fact that Fc receptor activation may be involved in rapid NETosis. Increase in phagocytosis as well as NET formation using C3b-coated beads in comparison to uncoated beads, may be another sign that NETosis and phagocytosis are collaborating, although one may not be the prerequisite of the other phenomenon.

### Rapid NET formation is NADPH oxidase-independent 

**Figure 6:**
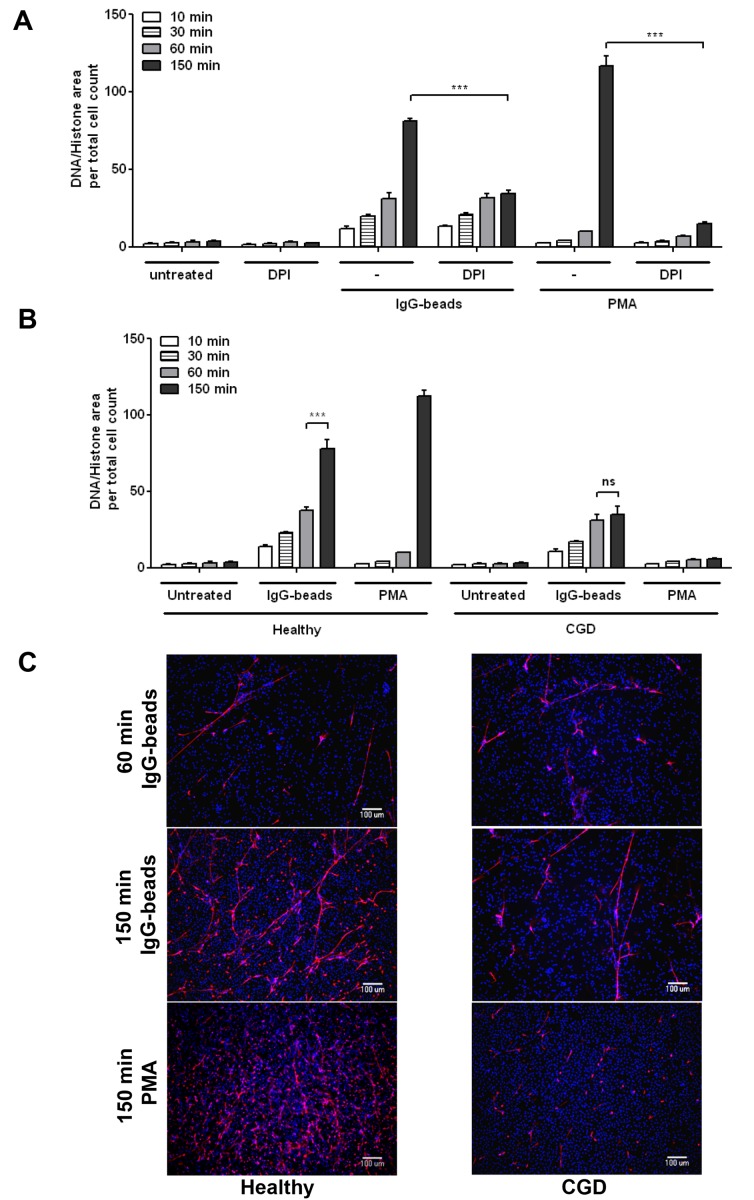
Rapid NETosis induced by IgG-beads is NADPH-oxidase independent (A) Human neutrophils from healthy donors were pre-treated with DPI for 30 min, followed by incubation of cells with IgG-beads or PMA for 10, 30, 60 and 150 min. Note that DPI could not decrease early NETosis, whereas late NETosis (150 min) induced by either PMA or IgG-beads decreased by DPI pre-treatment. (B) Human neutrophils from healthy donors as well as from CGD patients were treated with IgG-beads or PMA for 10 to 150 min, immunocytochemistry were performed to quantify NET generation. Note that CGD neutrophils were able to generate rapid NETs by IgG-beads but were not capable of producing NETs with PMA. NET generation of CGD neutrophils induced by IgG-beads was not also increased in longer incubation times (150 min). (C) Representative immuno-cytochemistry analysis of neutrophils from healthy donor as well as CGD patient after stimulation of cells with IgG-beads or PMA. DNA Histone (red); Hoechst 33342 (blue).

To further investigate the characteristics of this type of NETosis induced by opsonized particles, we wondered whether this rapid NETosis, similar to classical slow PMA-induced NET formation, depends on NADPH oxidase function. Therefore, prior to stimulation, neutrophils were pre-incubated with DPI to chemically inhibit NADPH oxidase activity. DPI fully abolished PMA-induced NETosis; however, rapid NET formation, induced by IgG-beads, was not impaired in the presence of DPI (**Figure 6** [A]). Of note, late NET formation after 150 min was diminished with DPI treatment, suggesting that rapid, but not slow NET formation is NADPH oxidase-independent (**Figure 6** [A]). To rule out the possibility that DPI was incapable of inhibiting NADPH oxidase, we used neutrophils from chronic granulomatous disease (CGD) patients with a deficiency of the NADPH oxidase complex. Neutrophils from these CGD patients, similar to healthy donors, were able to form rapid NETs in the presence of IgG-beads, but NET formation was not further enhanced after 150 min of stimulation (**Figure 6** [B]), indicating that the NADPH oxidase is dispensable for rapid NET formation. Other studies have recently shown that NETs can also be formed independent of NADPH oxidase, for instance when induced by *S. aureus*, singlet oxygen or glucan in the presence of fibronectin^19,20,31^. Also, Chen and colleagues have demonstrated that soluble immune complexes were able to form NETs through a process that did not require NADPH oxidase, myeloperoxidase or neutrophil elastase^32^. In the study performed by Chen et al, they checked endocytosis of soluble immune complex (BSA/anti-BSA), and the authors claimed that NET formation by this immune complex is selective, as phagocytosis of IgG-opsonized RBC did not induce NET. However, we could observe clearly NET formation by IgG or C3b-coated beads. Therefore, it seems again the type of stimulus has a key role in induction of NET formation, and as recently suggested, the requirement of NADPH oxidase for NETosis may vary depending on the stimulus^33-35^. Moreover, using a very sensitive method to follow ROS (EPR), total as well as intracellular superoxide generation was measured (**Supplementary Figure [S2]**). As expected, during the course of incubation there was a significant increase in ROS generation when cells treated with PMA, whereas only a slight increase in ROS generation was observed when IgG-beads were used as stimuli. Therefore, rapid NET formation induced by IgG-beads is not only NADPH-oxidase independent, but it seems that superoxide generation from sources other than NADPH-oxidase is dispensable for rapid NETosis.

In search for a cooperative effect of phagocytosis on NETosis, we found that opsonized particles IgG- or C3b-coated beads, as phagocytosis inducers, triggered rapid NET formation. By blocking the binding of opsonized particles to the FcγR, not only phagocytosis but also NET generation was decreased. Since phagocytosis alone as a process of 'chasing and hunting' individual bacteria appears to be ineffective to remove the rapidly disseminating microbial invaders extracellularly, rapid NETosis may support phagocytosis of bacteria in the neutrophil’s microenvironment. To be efficient in combating infections, it is likely that neutrophils use different strategies and mechanisms for slow and rapid NET formation. However, secretion of bacterial products and enzymes, for instance, DNase or FcγR antagonist might be strategies employed by microorganisms to escape from the host defense mechanisms. The ratio of host anti-microbial factors and the efficiency of total defense mechanisms versus the strategies that microorganisms have adapted to escape from the innate and adaptive immunity are probably the key factors which identify the destiny of host-microorganism battle. How neutrophils decide which pathways to choose is still unsolved. Modulating rapid versus slow NET formation may represent promising therapeutic strategies for infectious or auto-inflammatory diseases.
