# Supplementary material for: Characterization of rapid neutrophil extracellular trap formation and its cooperation with phagocytosis in human neutrophils
Source: Discoveries (Craiova). 2014 Jun 30;2(2):e19. doi: 10.15190/d.2014.11 (PMC6941580; doi:10.15190/d.2014.11)
Supplement: Supplementary file 1 [file discoveries-02-019-s001.pdf]

Discoveries Journals
publishing the future
DISCOVERIES 2014, Apr-Jun, 2(2): e19
DOI: 10.15190/d.2014.11

Supplementary Figures

Characterization of rapid neutrophil extracellular trap formation and its cooperation with phagocytosis in human neutrophils

Mona Saffarzadeh, Hector A. Cabrera-Fuentes, Florian Veit, Dongsheng Jiang, Karin Scharffetter-Kochanek, Christian Gille, Suzan H. M. Rooijakkers, Dominik Hartl, Klaus T. Preissner*

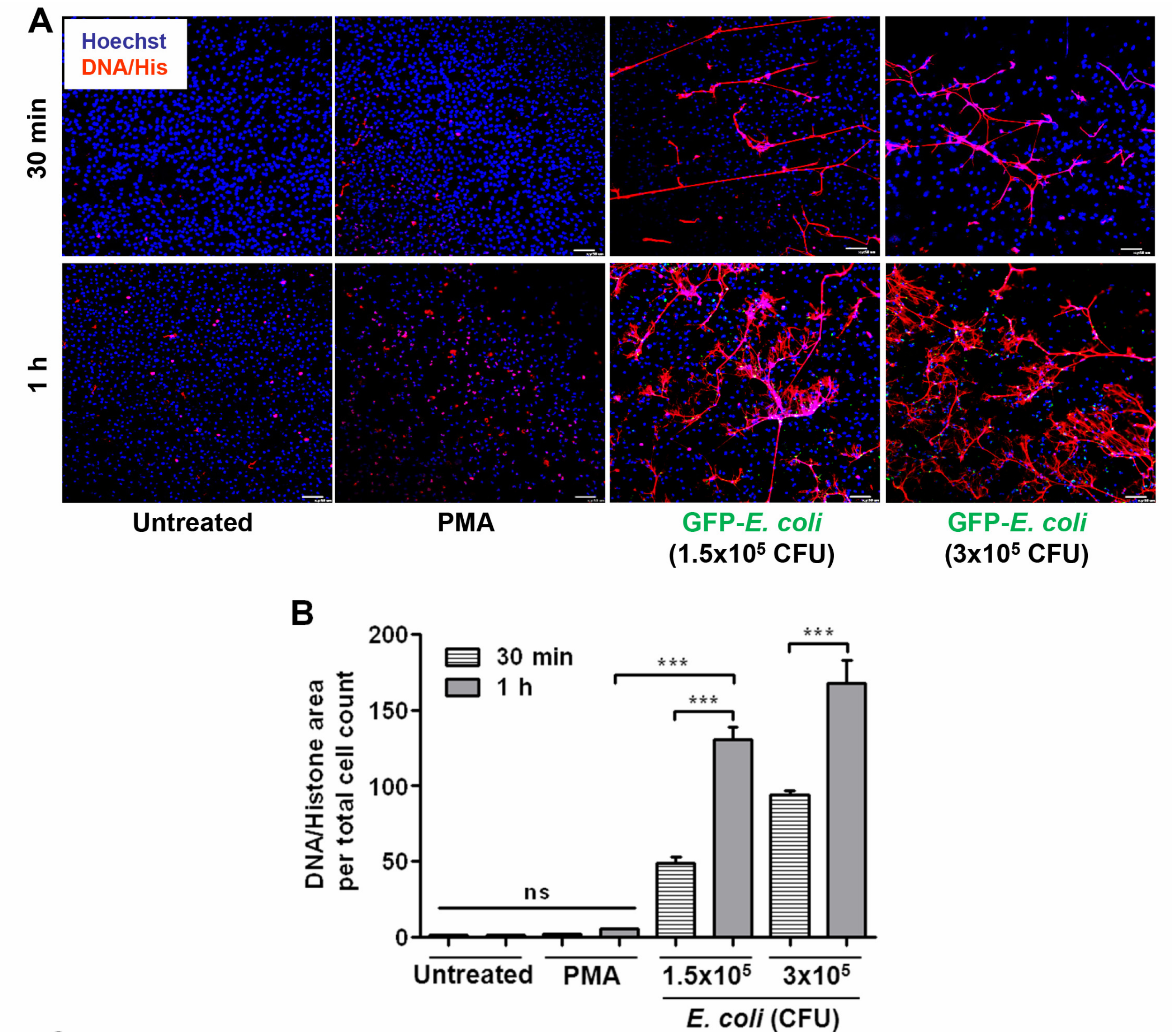

Figure S1. ***E. coli*** **accelerates NET formation**
(A) Human neutrophils were incubated with PMA or *E. coli*-GFP (*Escherichia coli* DH5α, carrying the green fluorescent protein (GFP)-mut2 encoding plasmid pCD353) for 30 min or 1 h, and immunocytochemistry was performed to detect NETosis which is depicted by DNA histone antibody. Nuclear stain: Hoechst 33342, scale bar: 50 µm. Note that PMA alone did not induce considerable NET formation during 1 h, while *E. coli* accelerates NETosis. (B) NET formation was induced by PMA or *E. coli* for 30 min or 1 h, and NETosis was quantified by ratio of DNA-histone area per number of cells as analyzed in panel A.

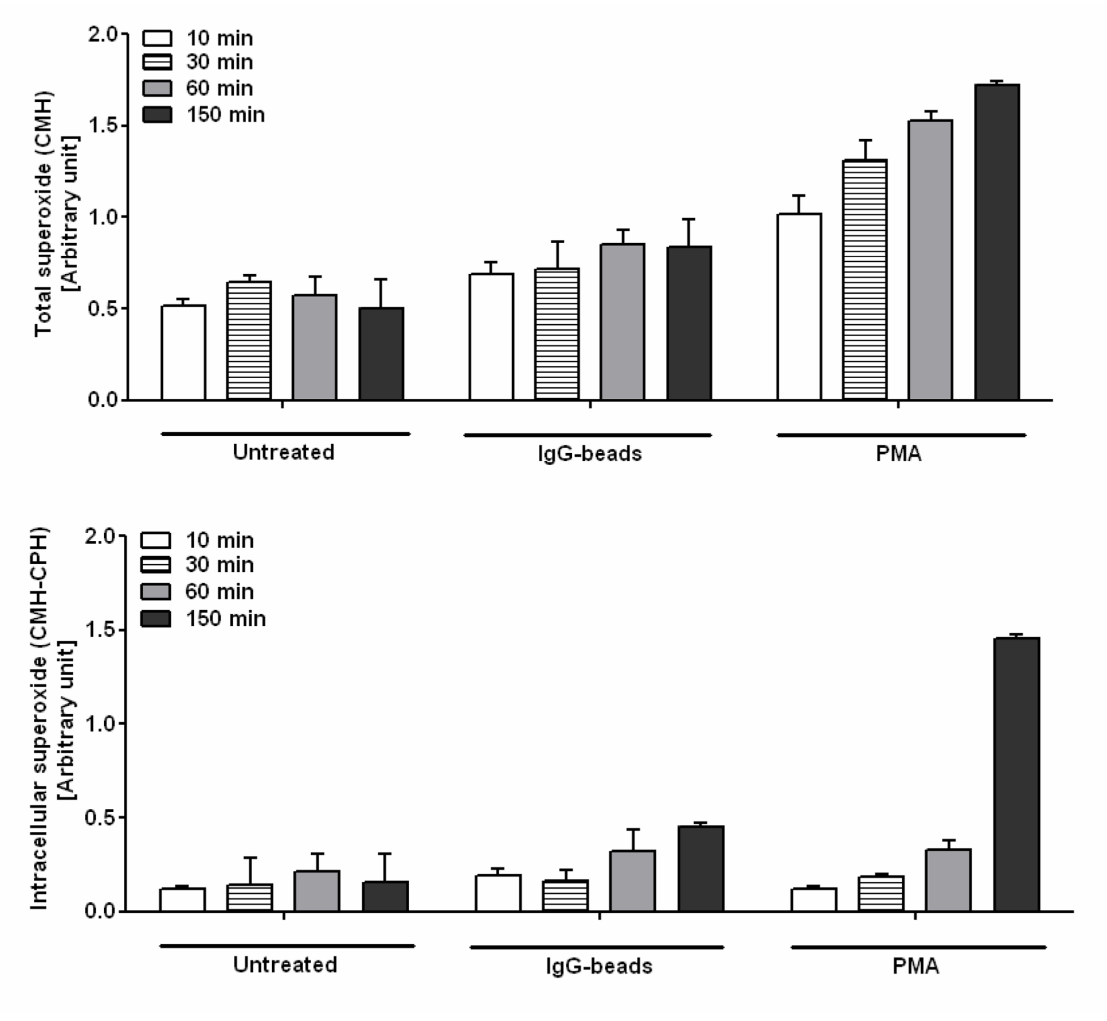

Figure S2. Kinetics of superoxide generation using EPR measurements
Neutrophils were incubated with IgG-beads or PMA for 10, 30, 60 and 150 min. Thereafter, cells were treated with CMH or CPH, shock-frozen and ROS generation was measured using ESR spectrometer. (A) Total ROS was measured using CMH which detects intra and extracellular ROS. (B) Intracellular ROS was calculated by subtracting the total ROS from the extracellular ROS (which was measured by CPH). Superoxide generation was depicted as arbitrary unit.
